# Impact of Education, Sex, and Residence on Tinnitus Distress, Depression, and Anxiety

**DOI:** 10.3390/audiolres16030078

**Published:** 2026-05-22

**Authors:** András Molnár, Panayiota Mavrogeni, Stefani Maihoub

**Affiliations:** 1 Opera Clinic, Protone Audio Kft., Lázár u 4, 1065 Budapest, Hungary; 2 Faculty of Health Sciences Doctoral School, University of Pécs, Vörösmarty Street 4, 7621 Pécs, Hungary; mavrogeni.panayiota@edu.pte.hu; 3 Maihoub ENT Clinic, Aliakmona Street 16, 3117 Limassol, Cyprus; stephaniemaihoub@gmail.com

**Keywords:** tinnitus, tinnitus handicap, depression, anxiety, residence, educational level, sex

## Abstract

**Objectives:** This study aimed to analyse the effects of educational levels, sex, and residence on tinnitus-related distress, as well as the severity of depression and anxiety. **Material and methods:** A total of 235 patients with primary subjective tinnitus participated in the study. These patients underwent thorough evaluations in otorhinolaryngology and audiology. Additionally, all patients completed the Tinnitus Handicap Inventory (THI), Beck Depression Inventory (BDI), and the Generalised Anxiety Disorder-7 (GAD-7) questionnaires. **Results:** Patients with a primary school education scored significantly higher on the functional (*F*) subscale of the THI. When examining depression and anxiety levels, it was observed that patients with a primary school education exhibited the highest levels of anxiety, whereas those with a university education displayed the highest levels of depression. When analysing the effects of residency, slightly lower total THI scores were observed in individuals living in metropolitan areas. When comparing the subscale results of the THI, patients residing in metropolitan areas exhibited significantly lower scores on the *F* subscale. In terms of the total BDI and GAD-7 scores, there were no statistically significant differences observed. Women had slightly higher scores on the BDI and GAD-7, without statistical differences. However, women had significantly higher total THI scores. Additionally, women exhibited statistically significantly higher scores on the catastrophic (*C)* subscale. However, the results for the *F* and emotional (*E*) subscales did not show any statistically significant differences. There was no correlation between age and the THI, BDI, or GAD-7 scores. **Conclusions:** The results of this study reveal significant differences in tinnitus distress based on sex, educational levels, and residence locations, along with the presence of psychiatric symptoms, which should also be considered in tinnitus management.

## 1. Introduction

Tinnitus refers to the perception of sound in the absence of an external sound stimulus. It is a common condition worldwide and can significantly impact an individual’s quality of life [1]. Tinnitus may affect between 5% and 43% of the global population [2]. Tinnitus often co-occurs with psychological issues such as depression and anxiety [3]. A previous study reported a median prevalence of 33% for depression in patients with tinnitus [4], while anxiety generally ranged from 28% to 45% [5]. Additionally, earlier research indicated a pooled prevalence of suicidal ideation in individuals with tinnitus at 20.6 per cent [6]. While many cases of tinnitus are linked to otological conditions, particularly sensorineural hearing loss, it is important to also consider systemic factors that may contribute [7]. In some instances, however, a specific underlying cause of tinnitus may not be identifiable. The pooled prevalence of diagnosed tinnitus was reported to be low at 3.4% [8].

Epidemiological factors can significantly affect tinnitus, particularly whether it becomes bothersome in some individuals but not in others. Previous studies on the impact of epidemiological factors on tinnitus have yielded conflicting results. Some studies have reported a slight predominance of females in terms of sex, particularly concerning more distressing tinnitus [8]. However, some reports have found no clear evidence of a difference in tinnitus between sexes [9]. With aging, there is an increased risk of developing tinnitus [10], which can also be attributed to presbycusis, a condition where sensorineural hearing loss further heightens the risk of tinnitus [11]. Previous research on a Korean sample found that low education levels were linked to moderate to severe tinnitus [12]. However, there have been few studies specifically addressing how educational levels contribute to tinnitus distress. Some studies have highlighted the impact of residential location on transportation noise and the risk of tinnitus [13]. Others have noted that higher rates of tinnitus occur in rural areas [14]. However, there is still limited data on how residential location contributes to differences in tinnitus prevalence.

The current study aims to analyse the potential effects of demographic factors on the severity of tinnitus and the presence of co-occurring depression and anxiety. This analysis, primarily based on descriptive statistics, will examine various parameters, including age, sex, education levels, and residential areas. These insights will help us better understand the factors that contribute to more distressing tinnitus.

## 2. Materials and Methods

### 2.1. Study Population

**Figure 1:**
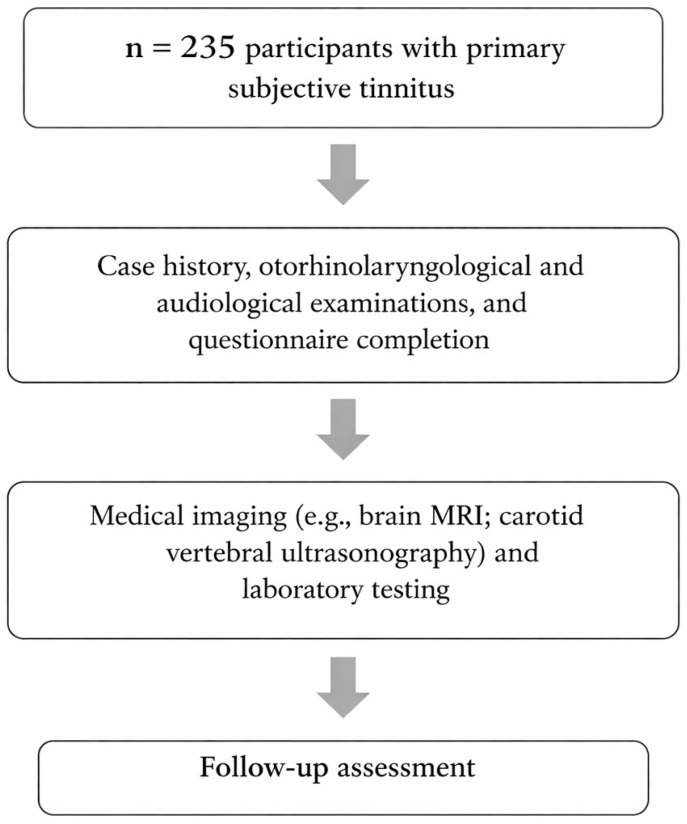
A flowchart illustrating the study process. MRI = magnetic resonance imaging.

A total of 235 participants with primary subjective tinnitus were enrolled in this study. The flowchart in Figure 1 illustrates the investigation process. The participants in this investigation have been assessed in a clinical setting. Each participant received comprehensive clinical management from a specialist experienced in tinnitus treatment, which included general otorhinolaryngological examinations, audiological assessments, detailed tests (such as brain MRI, carotid and vertebral ultrasonography, and laboratory tests), as well as self-reported questionnaires, as outlined below. Tinnitus cases were classified as acute or chronic, based on whether the symptoms started within the last 3 months or earlier. The criteria for including participants in the study were as follows: individuals must have experienced primary subjective persistent tinnitus for at least two weeks (which includes both acute and chronic cases), be over 18 years of age, provide consent to participate in the investigation, and have all necessary clinical and demographic data available. Exclusion criteria included secondary cases of tinnitus, any previous treatments for tinnitus, and previously diagnosed psychiatric disorders or treatments. Information regarding residence has been collected from medical records using the permanent address provided by the participants during the registration process. During the examination, the patients were asked to confirm whether the correct address had been recorded. The educational levels were recorded based on the highest degree attained by the patients, a detail routinely collected during case history taking. All participants provided written consent to take part in the study. The investigation adhered to the Declaration of Helsinki and received approval from the Hungarian ETT TUKEB (approval number: BM/29864–1/2024, approval date: 9 December 2024).

### 2.2. Questionnaires

#### 2.2.1. Tinnitus Handicap Inventory (THI)

The THI analyses the impact of tinnitus on daily functioning, focusing on three main scales: functional (*F*), emotional (*E*), and catastrophic (*C*). The functional scale assesses aspects such as social interactions and daily activities, the emotional scale relates to feelings like depression and anxiety, and the catastrophic scale addresses feelings of loss of control. Patients answer each question with ‘yes’ (4 points), ‘sometimes’ (2 points), or ‘no’ (0 points). The total scores of the THI can be calculated by summing the points from the three scales. According to the total THI, tinnitus can be categorised into five levels: no handicap (0–16 points), mild handicap (18–36 points), moderate handicap (38–56 points), severe handicap (58–76 points), and catastrophic handicap (78–100 points) [15]. Participants completed the THI in Hungarian.

#### 2.2.2. Generalised Anxiety Disorder-7 (GAD-7)

To analyse the co-occurrence of anxiety, all participants completed the GAD-7 questionnaire. The GAD-7 consists of 7 items designed to measure levels of anxiety and worry. Each question can be answered using a 4-point Likert scale, with response options as follows: 0 (‘not at all’), 1 (‘several days’), 2 (‘more than half the days’), and 3 (‘nearly every day’). Scores of 0–4 indicate minimal or no anxiety, 5–9 points represent mild anxiety, 10–14 points denote moderate anxiety, and 15–21 points signify severe anxiety [16]. The GAD-7 showed a strong internal consistency [17]. Participants filled out the GAD-7 questionnaire in Hungarian.

#### 2.2.3. Beck Depression Inventory (BDI)

To assess the severity of depressive symptoms, the shortened 13-item version of the BDI was used, which has been shown to be a reliable questionnaire for a Hungarian sample [18]. Participants respond by selecting a score from 0 (‘not at all’) to 3 (‘very much’), with a maximum total score of 39. A score of 0–5 indicates no depressive symptoms, scores of 6–11 suggest mild depression, scores of 12–15 indicate moderately severe depression, and scores of 16 and above indicate severe depression. The BDI demonstrated strong internal consistency and reliability [19].

The questionnaires utilised in this investigation have been previously validated and demonstrated strong internal consistency.

### 2.3. Statistical Analysis

The data processing in this investigation primarily relied on descriptive statistics. Data processing was conducted using IBM SPSS version 25 software (IBM Corporation, Armonk, NY, USA). Outliers have been considered appropriately in each case, with data points not representing the study population removed. Outliers were retained when seen as a natural variation, and corrections were made if necessary. Additionally, sensitivity analysis was conducted, first including outliers and then excluding them. The Shapiro–Wilk test was employed to assess the data’s distribution, revealing a normal distribution in most instances. Continuous variables were typically reported as means and standard deviations (SD) when normally distributed, or as medians and interquartile ranges (IQRs) when not. To compare the variables, both the *t*-test and One-Way ANOVA tests were utilised. The Chi-squared test was utilised for the categorical analysis. For analysing potential correlations, the Pearson correlation test was applied. A significance level of *p* < 0.05 was consistently applied across all analyses.

## 3. Results

The study population’s basic characteristics are presented in Table 1.

According to Table 1, the peak age for developing tinnitus is around 50 years, with a slight predominance in females. Tinnitus was primarily reported as bilateral in 46.4% of cases and left-sided in 29.8%. 32% of participants reported acute tinnitus, while the rest experienced chronic tinnitus. Most patients had either a secondary school education (33.6%) or a university degree (59.1%). Additionally, patients were mostly located in the capital city or the metropolitan area based on their primary residence. In the study sample based on primary residences, no significant differences were found when comparing ages (*p* = 0.357, *F* = 3.23, One-Way ANOVA test) and sexes (*p* = 0.23, Cramér’s *V* = 0.11, Chi-squared test). Additionally, no significant differences were observed when comparing the time intervals since the onset of symptoms (*p* = 0.879, *F* = 0.67) and the highest educational levels (*p* = 0.22, Cramér’s *V* = 0.133) based on primary residences. These results confirm that the groups are statistically comparable.

**Figure 2:**
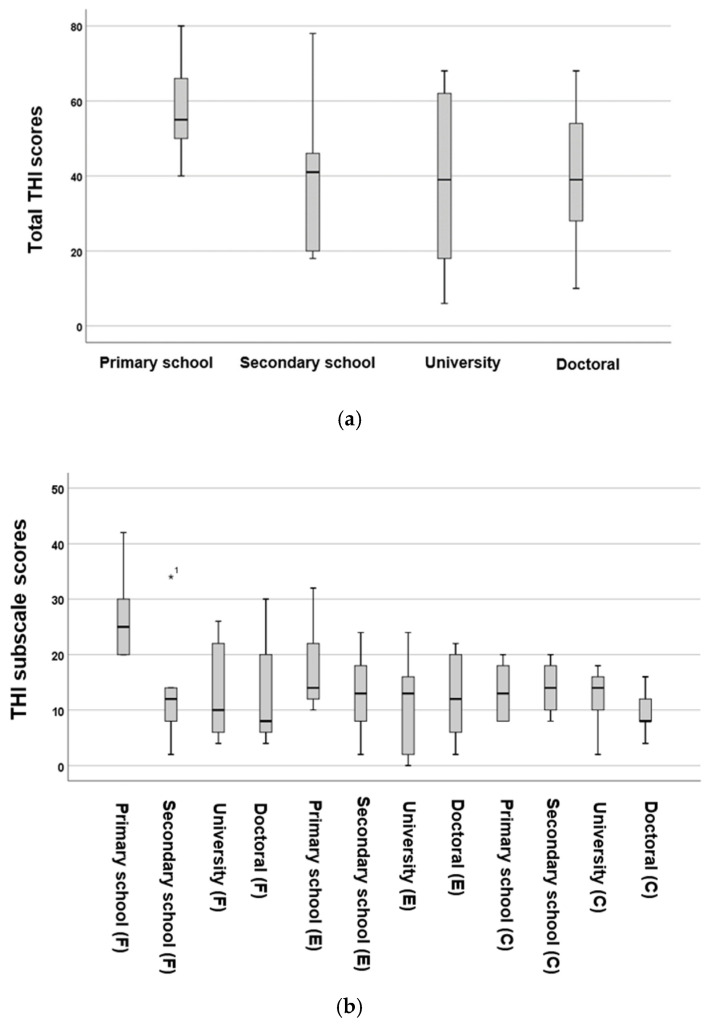
Boxplots illustrating the distributions of total THI scores (**a**) and their subscale (**b**) scores across various educational level groups. The boxes represent the middle 50% of the data, while the whiskers indicate the upper and lower 25%. The black line that divides each box denotes the median values. *C* = catastrophic, *E* = emotional, *F* = functional, THI = Tinnitus Handicap Inventory. Statistical analysis was conducted using the One-Way ANOVA test (*p* < 0.05 *). The asterisk and number depict the outlier.

**Figure 3:**
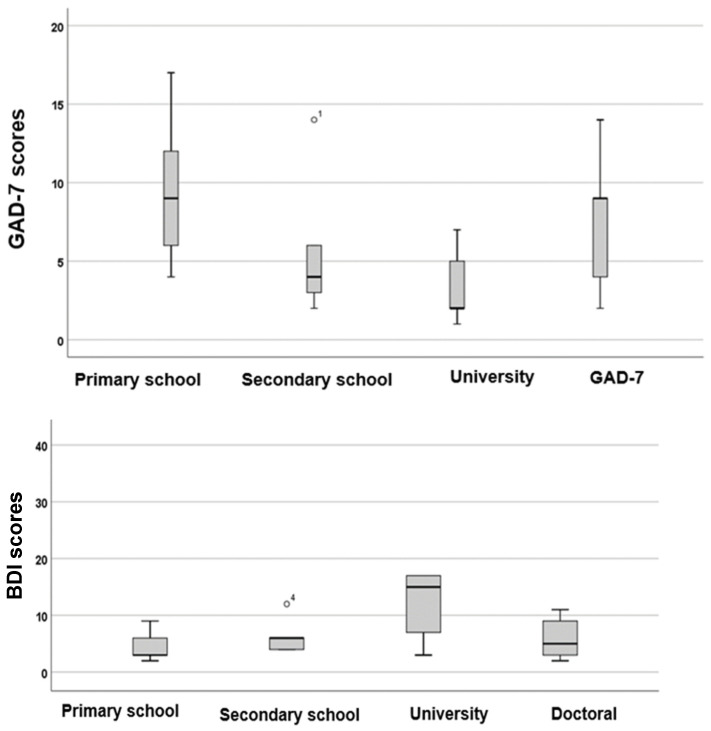
Boxplots showing the distributions of total GAD-7 and BDI scores for different educational level groups. The boxes represent the middle 50% of the data, while the whiskers indicate the upper and lower 25%. The black line that divides each box denotes the median values. BDI = Beck Depression Inventory, GAD-7 = Generalised Anxiety Disorder-7. Statistical analysis was conducted using the One-Way ANOVA test (*p* < 0.05). The circle and number indicate the outlier.

The total scores for THI, BDI, and GAD-7 were analysed based on the highest educational levels, as shown in Figure 2 and Figure 3.

The highest total THI scores were observed in patients whose highest level of education was primary school, with no statistically significant differences in total THI scores among the four groups (*p* = 0.951, *F* = 0.34, One-Way ANOVA test). Patients with only primary school education scored significantly higher on the *F* subscale (*p* = 0.03 *, *F* = 2.99), while there were no significant differences in the *E* (*p* = 0.49, *F* = 0.79) and *C* (*p* = 0.44, *F* = 0.90) subscales.

According to Figure 3, patients with a primary school education displayed the highest levels of anxiety (*p* = 0.177, *F* = 1.7), while those with a university education exhibited the highest levels of depression (*p* = 0.96, *F* = 0.09). However, the statistical analysis indicates that these differences were not statistically significant.

**Figure 4:**
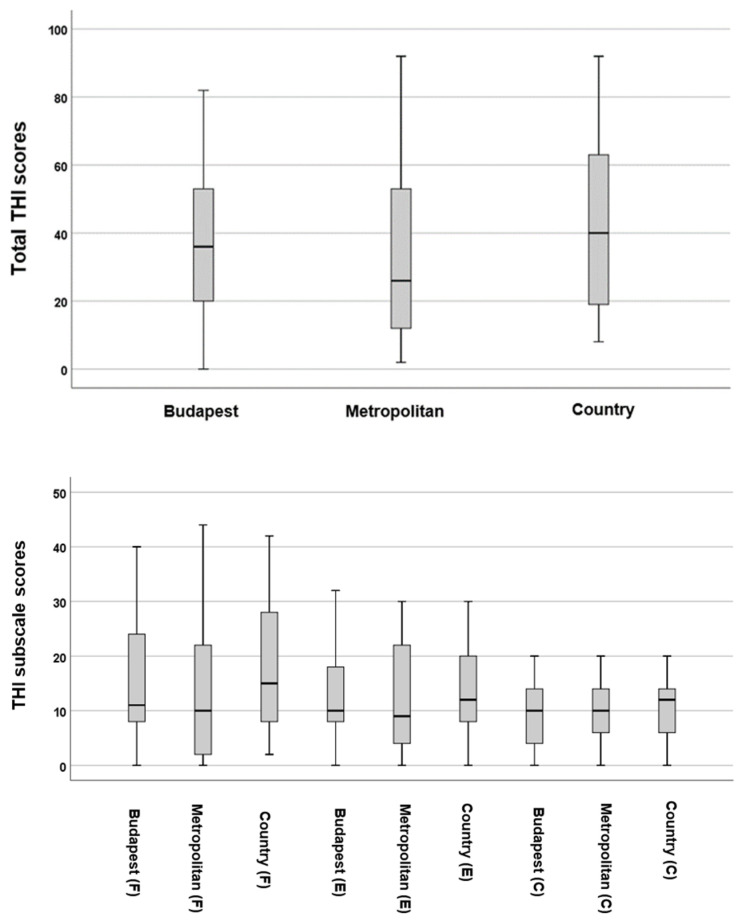
Boxplots illustrating the distribution of total THI scores across different types of residential areas. The boxes represent the middle 50% of the data, while the whiskers indicate the upper and lower 25%. The black line that divides each box denotes the median values. *C* = catastrophic, *E* = emotional, *F* = functional, THI = Tinnitus Handicap Inventory. Statistical analysis was conducted using the One-Way ANOVA test (*p* < 0.05).

The differences in total THI scores based on residential areas were not statistically significant (*p* = 0.22, *F* = 2.12). However, when examining the subscale results of the THI, patients living in metropolitan areas showed significantly lower scores on the *F* subscale (*p* = 0.012 *, *t* = −2.53, Cohen’s *d* = 0.43). In contrast, the *E* subscale (*p* = 0.08, *F* = 2.52) and the *C* subscale (*p* = 0.44, *F* = 0.81) did not show any statistically significant differences (Figure 4).

**Figure 5:**
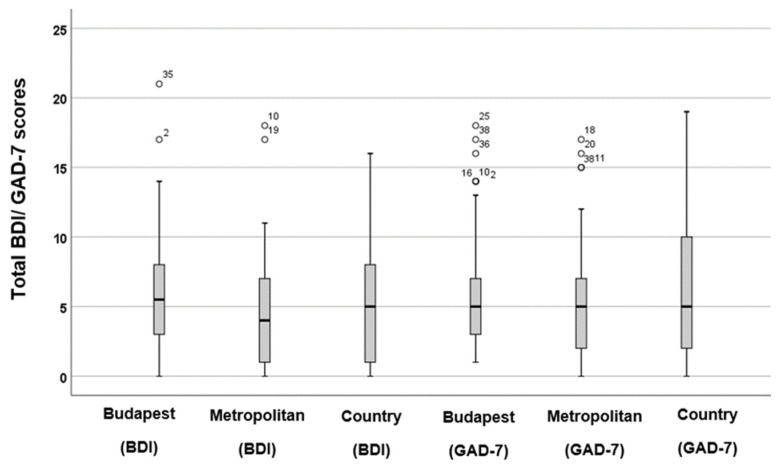
Boxplots illustrating the distribution of total BDI and GAD-7 scores across various types of residential areas. The boxes represent the middle 50% of the data, while the whiskers indicate the upper and lower 25%. The black line that divides each box denotes the median values. BDI = Beck Depression Inventory, GAD-7 = Generalised Anxiety Disorder-7. Statistical analysis was conducted using the One-Way ANOVA test (*p* < 0.05). The circles and numbers depict the outliers.

When comparing the BDI and GAD-7 scores among different groups (see Figure 5), slightly lower scores were observed for both the BDI (*p* = 0.96, *F* = 0.03) and GAD-7 (*p* = 0.07, *F* = 2.31) in metropolitan areas. However, these differences were not statistically significant.

**Figure 6:**
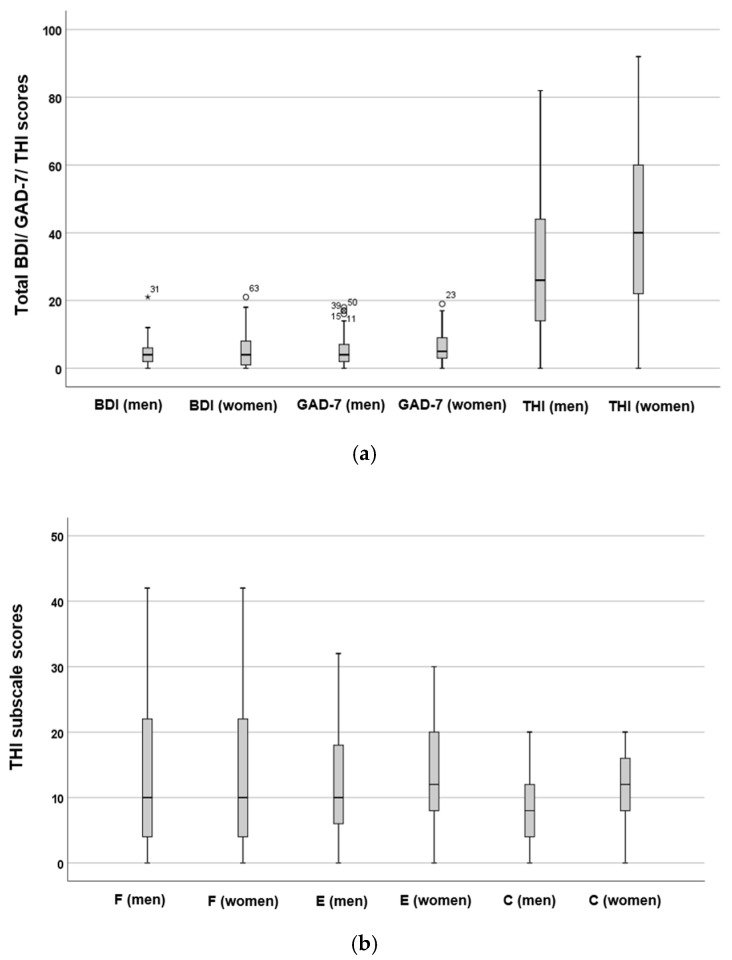
Total scores for the THI, BDI, and GAD-7 (**a**), as well as subscale scores (**b**) categorised by sex. The boxes represent the middle 50% of the data, while the whiskers indicate the upper and lower 25%. The black line that divides each box denotes the median values. BDI = Beck Depression Inventory, *C* = catastrophic, *E* = emotional, *F* = functional, GAD-7 = Generalised Anxiety Disorder-7, THI = Tinnitus Handicap Inventory. Statistical analysis was conducted using the *t*-test (*p* < 0.05 *). The numbers and circles depict the outliers.

Figure 6 depicts slightly higher BDI and GAD-7 scores for women; however, these differences were not statistically significant (*p* = 0.39, *t* = −0.27, Cohen’s *d* = 0.04). However, statistical analysis revealed that women had significantly higher total THI scores (*p* = 0.012 *, *t* = −2.24, Cohen’s *d* = 0.33). When comparing the subscales of the THI, the *F* (*p* = 0.47, t = −0.05, Cohen’s *d* = 0.01) and *E* (*p* = 0.35, *t* = 0.37, Cohen’s *d* = 0.06) subscale scores did not show statistically significant differences between sexes. However, women exhibited statistically significantly higher scores in the *C* subscale (*p* = 0.009 *, *t* = 2.37, Cohen’s *d* = 0.35).

To further analyse the correlations between depression, anxiety, and tinnitus, a correlation analysis was conducted, as shown in Table 2.

As summarised in Table 2, there was no significant correlation between age and total THI scores (rho = −0.004, *p* = 0.959), nor between age and the BDI scores (rho = −0.081, *p* = 0.268) or the GAD-7 scores (rho = −0.091, *p* = 0.211). However, significant positive correlations were found between total THI scores and BDI scores (rho = 0.347, *p* = 0.000 *) as well as between total THI scores and GAD-7 scores (rho = 0.400, *p* = 0.000 *). These correlations highlight the important relationships between tinnitus and the severity of psychiatric symptoms.

## 4. Discussion

This study analysed tinnitus distress severity and associated depression and anxiety based on sex, education level, and residential location. The main finding indicated that patients with a primary school education had statistically significantly higher scores on the *F* subscale of the THI. Analysis of the effect of residence showed significantly lower scores on the *F* subscale of the THI, indicating lower *F* subscale scores for individuals living in metropolitan areas. Furthermore, total THI scores and the *C* subscale scores were significantly higher for women. The results underscore the complexity of tinnitus severity, emphasising the need for targeted management strategies that consider various contributing factors.

This study found that women experience significantly greater tinnitus-related handicaps, particularly in terms of catastrophic attitudes. Several previous studies have compared sex differences in tinnitus. One notable investigation identified more risk factors for tinnitus in women. Additionally, sex has been recognised as a risk factor for developing tinnitus [8]. Research indicates that men and women have different responses to various treatment options. Women show a stronger reaction to high-definition transcranial direct current stimulation, whereas men tend to benefit more from tinnitus retraining therapy when combined with cognitive behavioural therapy [20]. Furthermore, sex-based differences in suicide attempts were noted, with severe tinnitus linked to attempts in women but not in men [21]. One investigation found that women reported increased tinnitus loudness in response to stress compared to men [22]. The differences in tinnitus-related distress may also be influenced by hormonal factors. Previous studies have highlighted the roles of both oestrogen and progesterone in the development of tinnitus [23]. These results highlight conflicting evidence regarding sex-related differences in tinnitus, underscoring the need for further research to implement more targeted intervention options. Our results show that tinnitus distress is significantly higher in women. In particular, the scores for the *C* subscale were notably higher. This subscale includes questions about extreme distress, fear, and feelings of being overwhelmed by the condition, such as feeling trapped, fearing a serious illness, or losing control over the situation. Therefore, the scores in this subscale require special attention and targeted management strategies.

When examining the relationship between education level and tinnitus severity, patients with only a primary school education exhibited significantly higher scores on the *F* subscale of the THI. The *F* subscale focuses on daily activities and includes aspects such as household management, interpersonal relationships, and stress counselling. It also measures difficulties in falling asleep and feelings of constant fatigue. The effect of having only a primary school education on tinnitus-related distress is connected to several factors, including lower health literacy, fewer coping strategies, and limited access to healthcare. Socioeconomic factors also significantly contribute to these issues, such as the prevalence of physically demanding jobs, higher job insecurity, and increased exposure to chronic stress. Additionally, certain job demands can make these challenges even more difficult, such as working in noisy environments. Research on the potential impact of educational levels on tinnitus is relatively limited. One specific study found that a longer duration of education was linked to lower odds of tinnitus [24]. In another investigation, there were no differences in the highest education levels achieved between participants with and without tinnitus [25]. Given the strong link between tinnitus and mental health issues like depression and anxiety, it is crucial to examine this relationship. Previous investigations consistently show an inverse relationship: as educational levels decrease, the prevalence of depression and anxiety increases [26,27]. Given the evident relationship between depression, anxiety, and tinnitus [28], a bidirectional relationship may be suggested with respect to the potential impact of educational levels. In our study, we found no statistically significant differences in depression and anxiety scores among the various educational level groups.

When comparing total THI scores based on residence areas, no statistically significant differences were found. It is important to note that only the *F* subscale demonstrated statistically significant differences based on residential area; however, the total THI score did not show any significant differences. Research on how residence areas impact the severity of tinnitus is limited. A study indicated that tinnitus is more prevalent in rural areas than in urban areas [14]. In a separate investigation, living in rural areas was linked to tinnitus with an odds ratio of 1.22 [29]. Another study reported that tinnitus prevalence was significantly higher among urban residents compared to rural inhabitants [30].

Previous investigations analysing the differences in depression and anxiety severities based on residential areas have produced conflicting results [31]. A study revealed a slightly higher prevalence of depression among rural adults compared to their urban counterparts; however, when accounting for population characteristics, the likelihood of depression was similar across both groups [32]. A large community study comparing urban and rural differences found a lower risk of depression among rural dwellers, which may be attributed to a stronger sense of community belonging [33]. Recent meta-analysis found slightly higher rates of depression and anxiety among youth in urban areas compared to rural areas; however, these differences were not significant and high heterogeneity was reported [34].

Although our results are of interest, the study has certain limitations that should be acknowledged. First, the sample size was relatively small, particularly regarding the limited number of participants whose highest educational level was primary school. This restricts the generalisability of our findings. Additionally, multiple testing corrections were not performed for specific reasons. Furthermore, we did not analyse audiometric profiles, prior treatments, or potential cases of occupational or noise-induced hearing loss.

## 5. Conclusions

This investigation shows that individuals with lower educational attainment and females experience a greater burden of tinnitus, as indicated by specific domains and overall scores on the THI. These findings underscore the importance of individualised approaches to tinnitus management that take into account the characteristics of each patient. Additionally, the observed connections between THI scores and those from the BDI and the GAD-7 highlight the strong relationship between the severity of tinnitus and psychological distress, particularly in relation to depression and anxiety. Although these findings provide valuable insights, the limited statistically significant differences across subscales point to the need for further research to better understand these relationships.

## Figures and Tables

**Table 1:** The basic parameters of the study population. The parameters in the table are categorised by residential areas, including the capital city, metropolitan area, and rural regions. IQR = interquartile range, SD = standard deviation, Q1 = first quartile, Q3 = third quartile. * One-Way ANOVA test, ** Chi-squared test. The significance level was established at *p* < 0.05.

	Total Study Group	Capital City	Metropolitan Area	Country	*p*-Value
Age (mean ± SD years)	50.13 ± 11.23	49.31 ± 10.27	53.42 ± 11.34	49.47 ± 11.78	0.357 *
Sex (men/women)	93/142	30/71	14/59	13/48	0.23 **
Tinnitus location, *n* (%)					0.12 **
Right	56 (23.8%)	24 (23.7%)	17 (23.2%)	15 (24.5%)	
Left	70 (29.8%)	31 (30.6%)	22 (30.1%)	19 (31.1%)	
Bilateral	109 (46.4%)	46 (45.7%)	34 (46.7%)	27 (44.4%)	
Beginning of symptoms (median months, IQR; Q1–Q3)	12 (34; 2–36)	12 (34; 2–36)	12 (35; 2–37)	12 (36; 3–36)	0.879 *
Highest educational level, *n* (%)					0.22 **
Primary school	9 (3.8%)	3 (2.9%)	2 (2.7%)	4 (6.5%)	
Secondary school	79 (33.6%)	32 (31.7%)	24 (32.9%)	23 (37.7%)	
University	139 (59.1%)	60 (59.4)	47 (64.4%)	32 (52.4%)	
Doctoral	8 (3.5%)	6 (6%)	0 (0%)	2 (3.27%)	
Primary residence, *n* (%)					
Capital city (Budapest)	101 (43%)				
Metropolitan area	73 (31%)				
Country	61 (26%)				

**Table 2:** Results of the Pearson correlation test between various parameters (*p* < 0.05 *). BDI = Beck Depression Inventory, GAD-7 = Generalised Anxiety Disorder-7, THI = Tinnitus Handicap Inventory.

Parameter	Rho	*p*-Value
Age-THI	−0.004	0.959
Age-BDI	−0.081	0.268
Age-GAD-7	−0.091	0.211
THI-BDI	0.347	0.000 *
THI-GAD-7	0.400	0.000 *

## Data Availability

The original contributions presented in this study are included in the article. Further inquiries can be directed to the corresponding author.

## Abbreviations

The following abbreviations are used in this manuscript:

BDI	Beck Depression Inventory
C	Catastrophic
E	Emotional
F	Functional
GABA	Gamma-aminobutyric acid
GAD-7	Generalised Anxiety Disorder-7
IBM	International Business Machines Corporation
IQR	Interquartile range
MRI	Magnetic resonance imaging
Q1	First quartile
Q3	Third quartile
SD	Standard deviation
SPSS	Statistical Package for the Social Sciences
THI	Tinnitus Handicap Inventory
